# Early clinical diagnosis of congenital insensitivity to pain with anhidrosis in an infant: a case report

**DOI:** 10.3389/fped.2026.1844909

**Published:** 2026-05-20

**Authors:** Ziqing Tang, Fenfen Xu, Piaopiao Zhang, Jing Zhang, Xinyu Kang, Hongyang Zhao

**Affiliations:** 1 School of Clinical Medicine, Shandong Second Medical University, Weifang, Shandong, China; 2 Department of Pediatrics, Jinan Central Hospital, Jinan, Shandong, China

**Keywords:** autosomal recessive disorder, case report, congenital insensitivity to pain with anhidrosis, *NTRK1* gene, TrkA protein

## Abstract

Congenital insensitivity to pain with anhidrosis (CIPA) is an extremely rare autosomal recessive disorder. Its core clinical manifestations include profound pain insensitivity, generalized anhidrosis, and subsequent recurrent hyperthermia. To date, only a few hundred cases have been reported in the literature. An 8-month-old female infant was admitted with a 14-day history of recurrent fever that was unresponsive to systemic antimicrobial therapy. Dynamic clinical monitoring revealed an environment-dependent body temperature. Physical examination showed dry and coarse palms, accompanied by painless oral ulcers and painless distal finger injuries. Retrospective history taking confirmed a lack of sweating during febrile episodes and an absence of pain perception. Whole-exome and Sanger sequencing identified compound heterozygous variants in the *NTRK1* gene, comprising a maternal intronic variant (c.851-33T > A) and a paternal large deletion at 1q23.1 encompassing exons 5–7. A definitive clinical diagnosis of CIPA was established. In infants presenting with refractory fever, clinicians should maintain a high index of suspicion for non-infectious etiologies. Early evaluation for anhidrosis and pain insensitivity, along with the inclusion of CIPA in the differential diagnosis, can expedite definitive diagnosis, prevent secondary injuries, and facilitate timely genetic counseling.

## 1 Introduction

The clinical phenotype of congenital insensitivity to pain with anhidrosis (CIPA) is highly insidious during infancy. Because infants cannot verbally communicate pain and anhidrosis is easily overlooked during routine physical examinations, unexplained recurrent fever often serves as the sole early clinical clue (1). Lacking other specific early manifestations, these patients are frequently misdiagnosed with refractory infections, leading to significant therapeutic delays. In this report, we detail the diagnostic workup of an 8-month-old infant with CIPA who initially presented with fever. This report aims to highlight the diagnostic value of dynamic temperature monitoring and provide practical clinical insights to improve the early recognition of this rare disorder in pediatric practice.

## 2 Case report

Chief Complaint: An 8-month-old girl presented to our pediatric department in February 2024 with a 14-day history of recurrent fever.

History of Present Illness: The fever started 14 days prior (Feb 10, 2024) without an apparent trigger, peaking at 39.8 °C. It would drop to normal but quickly rebound two to three times daily. She was initially diagnosed with stomatitis and a *mycoplasma infection* at a local hospital, where intravenous erythromycin was administered for two days (Feb 14–16) with poor clinical response. As her temperature continued to fluctuate between 37.2 °C and 39.6 °C, the regimen was adjusted to intravenous ceftriaxone, methylprednisolone, and bromhexine for six days (Feb 17–22). The fever remained refractory. She was subsequently transferred to our hospital on Feb 23, 2024.

Personal and Family History: She was her parents' first child, delivered at term via normal vaginal delivery with a birth weight of 3.1 kg. Although she could track objects visually at 1 month and hold her head steady at 4 months, her rolling milestone was delayed, and she currently cannot sit unsupported. Her parents denied consanguinity, and the family history was negative for congenital or genetic disorders.

**Figure 1:**
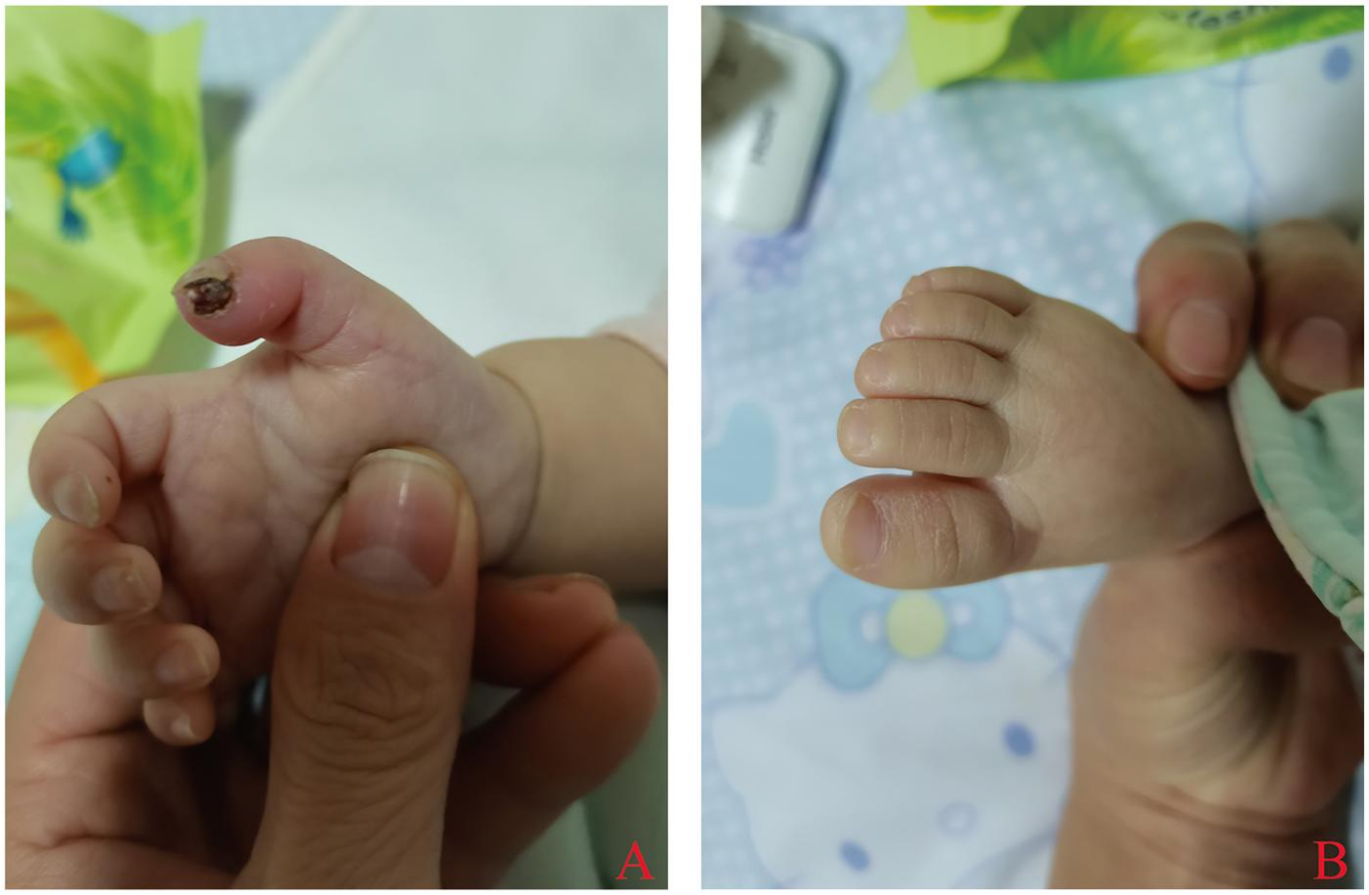
**(A)** Crusted lesion on the distal right thumb of the patient. **(B)** Dry and coarse skin on the right foot.

Physical Examination: Weight: 7 kg. Temperature: 39.3 °C. Blood pressure: 82/50 mmHg. Respiratory rate: 27 breaths/min. Heart rate: 130 beats/min. She was conscious with a fair general condition. Her pharynx was slightly hyperemic, but no gingival swelling or tongue ulcers were observed. Both palms were dry and coarse, with lesions and peeling affecting the distal right index finger and thumb (Figure 1). Cardiopulmonary and abdominal exams were unremarkable. Neurological assessment revealed mild generalized hypotonia and an inability to sit independently.

Clinical Course: Initial evaluation suggested an infectious etiology. Empiric cefoperazone/sulbactam and intravenous immunoglobulin (1 g/kg) were prescribed. Early tests showed a leukocyte count of 14.08 × 10^9^/L (68.3% lymphocytes) and hemoglobin at 99 g/L, while urinalysis, stool routine, serum biochemistry, CRP, and PCT were largely normal. The fever persisted despite treatment. After a cerebrospinal fluid (CSF) culture flagged Gram-positive cocci, antibiotic therapy was escalated to vancomycin. However, since CSF routine, glucose, chloride, and protein levels were normal, a repeat CSF culture was ordered to rule out sample contamination. This second culture returned negative, and vancomycin was discontinued (Feb 29–Mar 2). The peak temperature decreased slightly post-treatment, but the daily fever continued. Pathogen screening detected human rhinovirus nucleic acid, though her clinical trajectory did not match a typical self-limiting rhinovirus infection. With other pathogen panels returning negative, a non-infectious fever was suspected. Rheumatologic panels, immunoglobulins, lymphocyte subsets, and metabolic screens yielded no abnormalities.

**Figure 2:**
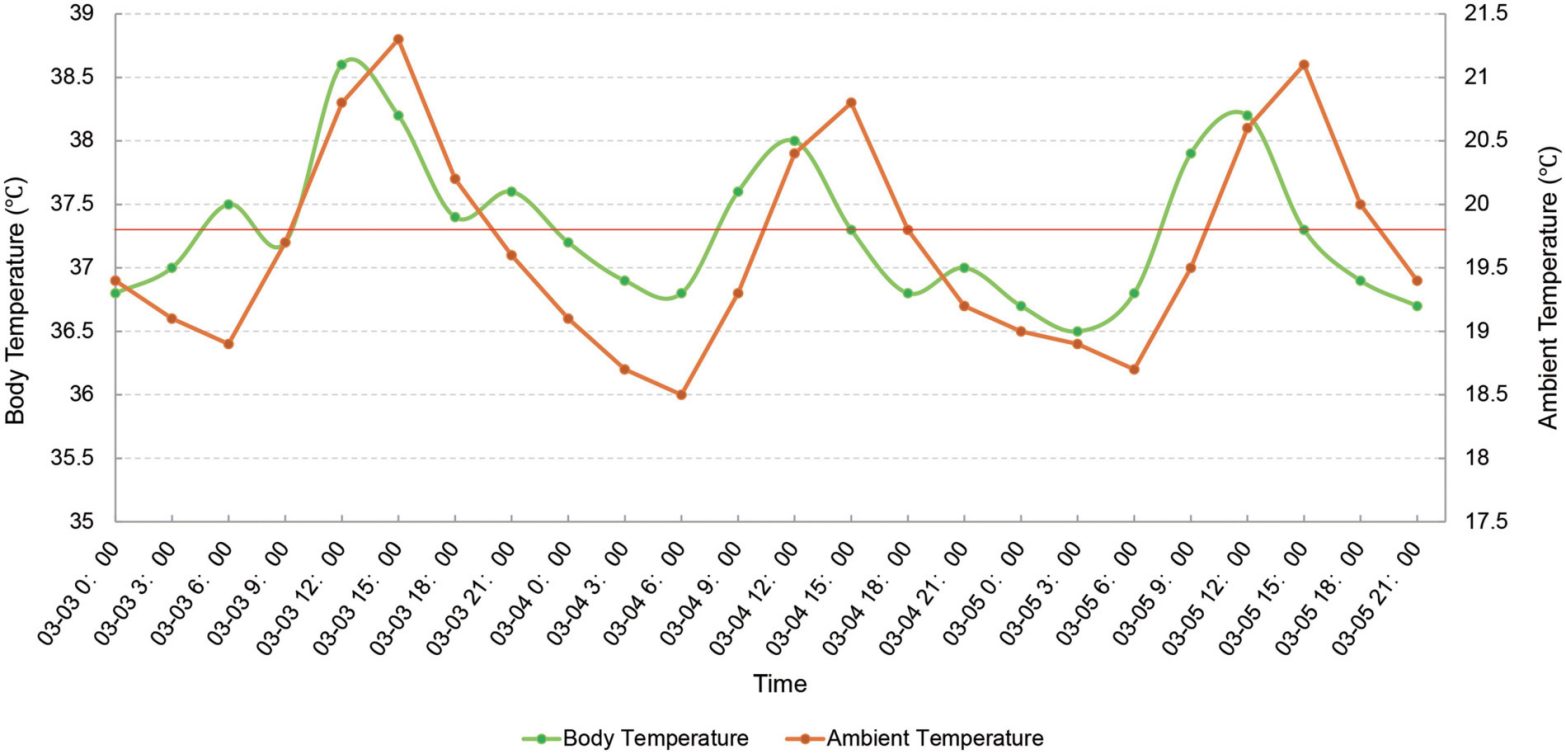
Continuous body temperature and concurrent ambient temperature curves of the patient following medication discontinuation (March 3–5, 2024). The horizontal red line indicates the threshold for normal body temperature (37.3 °C).

All antimicrobials and antipyretics were suspended on March 3, 2024, to clarify the fever pattern. Three-day intensive monitoring confirmed a distinct environmental dependency (Figure 2). Her basal temperature remained normal overnight and consistently rose in parallel with the daytime increase in ambient temperature; however, this synchrony was not perfectly maintained due to the timely implementation of physical cooling measures, such as unswaddling and reducing clothing. During hospitalization, she consumed overly hot food and developed a 0.5 cm burn blister on her tongue base. She showed no pain reflex from blister formation through its eventual mechanical rupture, and her feeding remained unaffected. Upon detailed retrospective review, this pain insensitivity dated back to early infancy. She only displayed instinctual body withdrawal rather than crying in pain during invasive procedures like venipuncture. Frequent biting of her fingertips during teething also failed to elicit distress signals. In addition, her parents recalled she never visibly sweated after taking ibuprofen for high fever. These unique clinical presentations prompted us to consider hereditary sensory and autonomic neuropathies (HSAN) and congenital developmental disorders. Although hypohidrotic ectodermal dysplasia (HED) also presents with infantile anhidrosis and recurrent environment-dependent fever, our patient lacked characteristic ectodermal defects (e.g., hypotrichosis), and her profound diminished pain perception was entirely inconsistent with HED. Within the HSAN spectrum, type I typically presents with adult-onset sensory neuropathy. Type II, despite its infantile onset, primarily affects sensory neurons and generally does not exhibit the complete generalized anhidrosis and recurrent hyperthermia observed in our case. Furthermore, while both HSAN III and V share features of pain insensitivity, HSAN III (familial dysautonomia) is characterized by defective lacrimation and hyperhidrosis rather than generalized anhidrosis, and patients with HSAN V maintain normal sweating and thermoregulatory functions. Having systematically ruled out these alternatives, we highly suspected CIPA as the primary clinical diagnosis (Table 1). The subsequent identification of compound heterozygous *NTRK1* mutations via whole-exome sequencing (WES) firmly corroborated our clinical hypothesis.

**Table 1:** Differential diagnosis of CIPA and other phenotypically related disorders.

Disease	Inheritance (Gene)	Age of Onset	Sensation	Sweating/Thermo	Cognition	Other Distinguishing Features
HSAN IV (CIPA)	AR (*NTRK1*)	Infancy	Profound pain/temp loss	Complete anhidrosis, recurrent fever	Intellectual disability	Self-mutilation, painless fractures, recurrent infections, joint deformities
HSAN I	AD (*SPTLC1*, etc.)	Adulthood	Distal pain/temp loss	Normal/distal hypohidrosis	Normal	Plantar ulcers, shooting pains
HSAN II	AR (*WNK1*, etc.)	Infancy/Childhood	Profound global loss	Variable hypohidrosis	Normal/mild impairment	Severe sensory ataxia, widespread areflexia, unnoticed traumatic injuries
HSAN III (Riley-Day)	AR (*ELP1*)	Infancy	Decreased pain/temp	Hyperhidrosis, erratic temp	Normal/mild impairment	Alacrima, absent fungiform papillae, episodic autonomic crises
HSAN V	AR (*NGF*)	Infancy/Childhood	Profound pain/temp loss	Normal	Normal	Neuropathic arthropathy (Charcot joints)
HED	XL/AR/AD (*EDA*, etc.)	Infancy	Normal	Hypohidrosis, recurrent fever	Normal	Hypodontia/anodontia, hypotrichosis, characteristic facial dysmorphism

**Figure 3:**
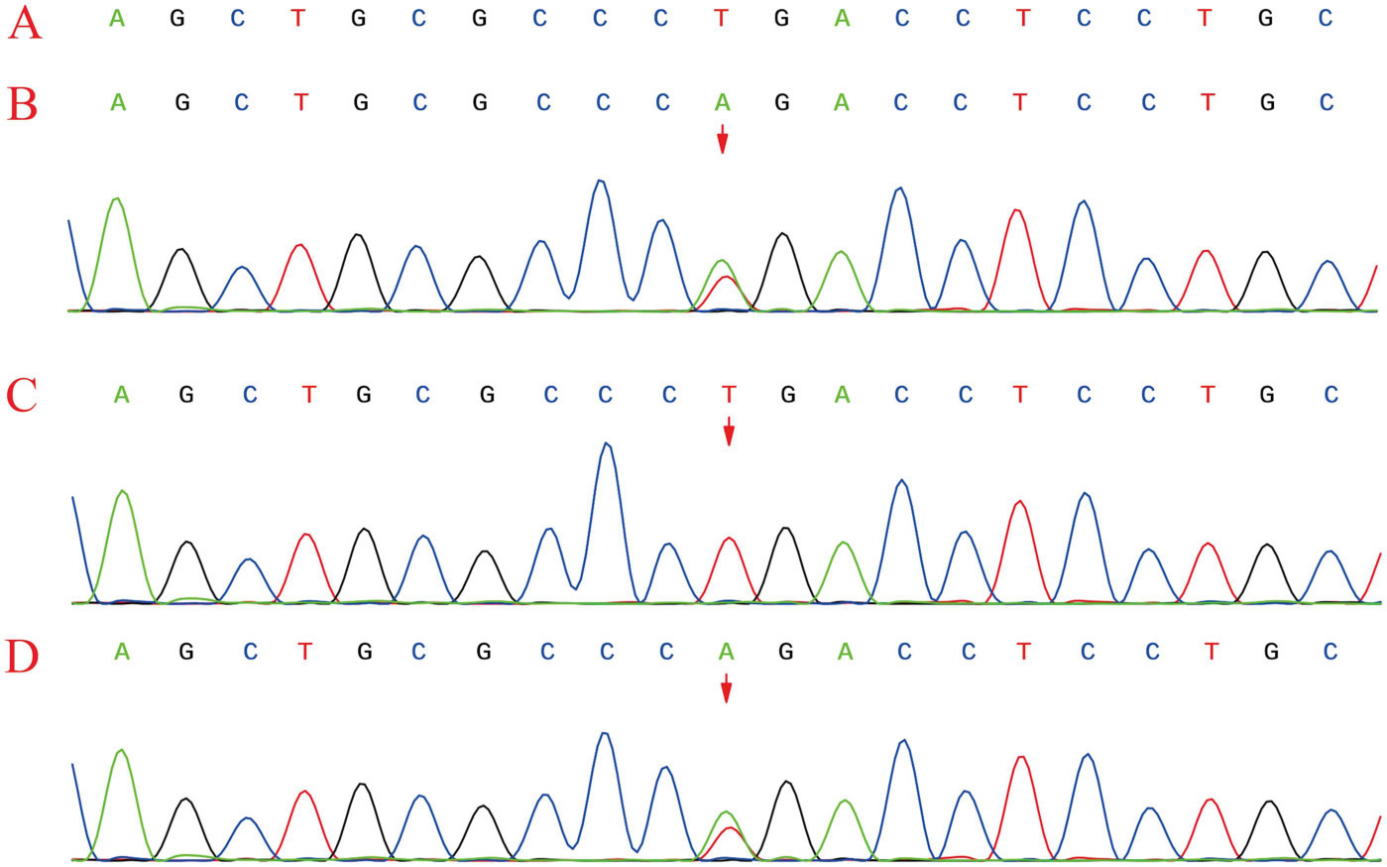
Sanger sequencing analysis of the *NTRK1* gene (NM_002529.4). The patient harbors a maternally inherited heterozygous intronic variant (c.851-33T > A). Chromatograms represent **(A)** the reference sequence, and the sequences of **(B)** the patient, **(C)** the father, and **(D)** the mother. Arrows indicate the mutated positions.

**Figure 4:**
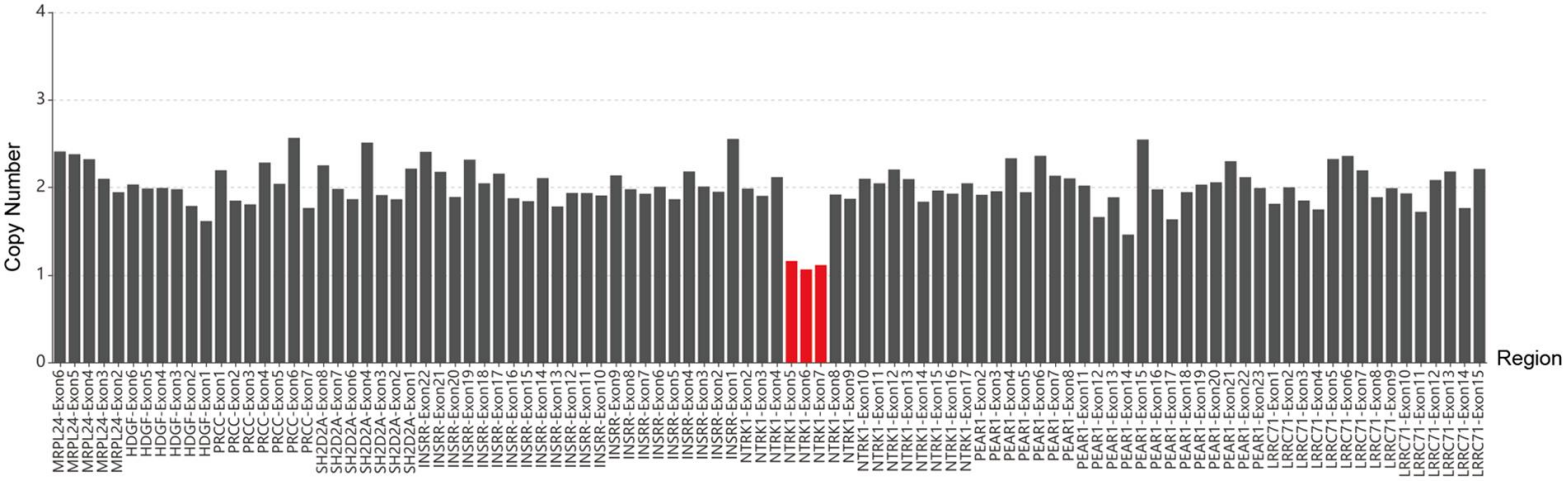
Genetic testing reveals a deletion variant in the *NTRK1* gene (NM_002529.4) of the patient.

Genetic Testing and Variant Analysis: WES and Sanger sequencing confirmed our clinical hypothesis, revealing that the infant carried compound heterozygous mutations in the *NTRK1* gene (NM_002529.4). Specifically, the infant harbored a maternally inherited c.851-33T > A variant (Figure 3). According to the American College of Medical Genetics and Genomics (ACMG) guidelines, this variant was classified as pathogenic (PVS1_Strong + PM2_Supporting + PM3_VeryStrong + PP1). Previous *in vitro* functional studies have established that this mutation activates an upstream cryptic splice acceptor site, resulting in the abnormal retention of a 137-bp intronic sequence in the mRNA (2). This frameshift mutation creates a premature stop codon at position 319, leading to a truncated tropomyosin receptor kinase A (TrkA) protein that lacks the crucial transmembrane and intracellular kinase domains, thereby entirely abolishing its signal transduction capacity (3). This experimentally validated splicing disruption mechanism was corroborated by our *in silico* analysis using SpliceAI (4), yielding a Delta Score for Acceptor Gain (DS_AG) of 0.47, a value substantially exceeding the conventional pathogenic threshold of 0.2 (Supplementary Figure 1A). Furthermore, using a WES-based copy number variation (CNV) analysis algorithm, a paternally inherited large deletion was identified in the 1q23.1 region, encompassing exons 5–7 of the *NTRK1* gene (Figure 4). Due to the exon-level resolution of the WES technique and the lack of probe coverage in deep intronic regions, the exact nucleotide breakpoints could not be precisely mapped (represented as chr1:?156837875-156841681?, estimated at 3.81 kb). Structural analysis using the Ensembl and UniProt databases revealed that exons 5–7 encode the leucine-rich repeat C-terminal domain (LRRCT) and the immunoglobulin-like C2-type 1 domain (5, 6), which represent the core functional regions for TrkA binding to nerve growth factor (NGF). Consequently, the deletion of this 422-bp segment not only causes a downstream frameshift but also directly abolishes these essential binding domains, rendering the TrkA protein completely inactive (Supplementary Figures 1B,C). Notably, although structural variants in *NTRK1* are rare, similar large deletions encompassing exons 5–7 have been previously documented and functionally validated as pathogenic variants in CIPA patients (7). In summary, these compound heterozygous mutations fundamentally clarify the pathogenic basis of congenital insensitivity to pain with anhidrosis in this patient.

Follow-up: The patient was discharged on March 6, 2024, with a home care plan prioritizing dynamic temperature monitoring, skin barrier maintenance, and injury prevention. At her most recent follow-up (approximately 2 years of age), despite having received no systematic rehabilitation training or therapeutic interventions, her gross and fine motor skills appeared age-appropriate based on the family's daily observations and comparisons with healthy peers. Furthermore, her language comprehension and expression were comparable to those of her peers, with the exception of mild articulation issues. She experienced no seizures or self-mutilation during the follow-up period. Although CIPA patients are prone to refractory fractures and delayed tissue healing, this patient exhibited normal local tissue repair. She sustained an accidental elbow fracture from a fall in March 2025, which was managed conservatively with a simple splint. The fracture healed uneventfully, with a healing timeframe comparable to that of healthy peers, resulting in full functional recovery of the affected limb.

## 3 Discussion

CIPA, a rare autosomal recessive disorder, was first linked to mutations in the *NTRK1* gene on chromosome 1 by Indo et al. in 1996 (8) This gene encodes TrkA, the primary receptor for NGF. TrkA defects disrupt NGF signaling and impair the development of sensory and sympathetic neurons (9). Recent studies indicate that the mutation types and specific sites within the *NTRK1* gene differentially affect TrkA protein function, which constitutes the molecular basis for the clinical phenotypic heterogeneity observed in CIPA patients (10). While certain mutations in less critical regions may retain residual receptor activity (11), those causing a complete loss of TrkA function typically produce a severe, classic CIPA phenotype. For instance, in the present case, the paternally inherited large deletion abolishes critical functional domains and causes a frameshift, rendering this allele completely inactive. Despite this variability, the hallmark features of CIPA remain consistent. Clinically, these patients present with a profound inability to perceive pain and an absence of sweating. Consequently, the combination of sensory deprivation and refractory hyperthermia frequently leads to severe complications, including self-mutilation and recurrent fractures. As the disease progresses, autonomic dysfunction disrupts tissue repair pathways, which compromises skin and bone healing (12). These deficits manifest as chronic ulcers, osteomyelitis, and Charcot neuroarthropathy. A subset of patients develops generalized dry skin, palmar hyperkeratosis, and hypotonia (13, 14). In the absence of consensus international guidelines, diagnosis typically hinges on the identification of hallmark clinical features, the exclusion of differential hereditary neuropathies, and ultimate molecular genetic confirmation. Clinical management focuses on symptom control and complication prevention, as current medicine offers no cure. Emerging therapies like gene editing and stem cell transplantation show promise in basic research, though their clinical translation requires further validation (15).

CIPA's insidious onset frequently causes diagnostic delays, especially in pre-verbal infants. The 8-month-old infant presented exclusively with recurrent high fever, and her temperature exhibited marked fluctuations despite standard systemic antibiotic therapy. This refractory fever, inconsistent with a typical infectious etiology, necessitated a broader diagnostic workup. The patient's lack of sweating during febrile episodes and her environment-dependent body temperature indicated a disruption of the efferent sympathetic pathways between the thermoregulatory center and peripheral target glands (16). Furthermore, her lack of distress during venipuncture, alongside painless mucosal and distal phalangeal injuries, provided definitive clinical evidence of sensory nerve interruption. The clinical constellation of refractory fever, impaired sweating, and insensitivity to pain ultimately led to the diagnosis of a hereditary sensory and autonomic neuropathy.

Several limitations of this study should be acknowledged. First, regarding the neurodevelopmental assessment, given the patient's young age, potential deficits in higher-order cognitive functions or complex learning abilities might not yet be fully manifest. This makes routine parental observations potentially insufficient to detect subtle developmental abnormalities; therefore, the lack of formal standardized neuropsychological evaluations (such as the Gesell or Bayley scales) remains a limitation. Second, regarding the genetic analysis, although WES failed to precisely map the exact nucleotide breakpoints of the paternally inherited large deletion, because the current results were already sufficient to establish a definitive diagnosis and formulate a clinical management plan, and due to the family's financial constraints, we did not utilize further advanced techniques (such as FISH or long-read sequencing) to address this technical limitation.

This case illustrates that astute clinical recognition remains central to the early diagnosis of CIPA. Because painless physical injuries and insidious anhidrosis frequently evade early detection, pediatricians must explicitly assess sweating function and pain reflexes in infants presenting with unexplained refractory fever. Incorporating these targeted clinical examinations into routine practice is crucial to minimize diagnostic delays and facilitate early genetic counseling.

## Data Availability

The original contributions presented in the study are included in the article/Supplementary Material, further inquiries can be directed to the corresponding author.
