# Supplementary material for: Early clinical diagnosis of congenital insensitivity to pain with anhidrosis in an infant: a case report
Source: Front Pediatr. 2026 May 20;14:1844909. doi: 10.3389/fped.2026.1844909 (PMC13230007; doi:10.3389/fped.2026.1844909)
Supplement: Supplementary file 1 [file Datasheet1.docx]

Supplementary Material

# Supplementary Figure


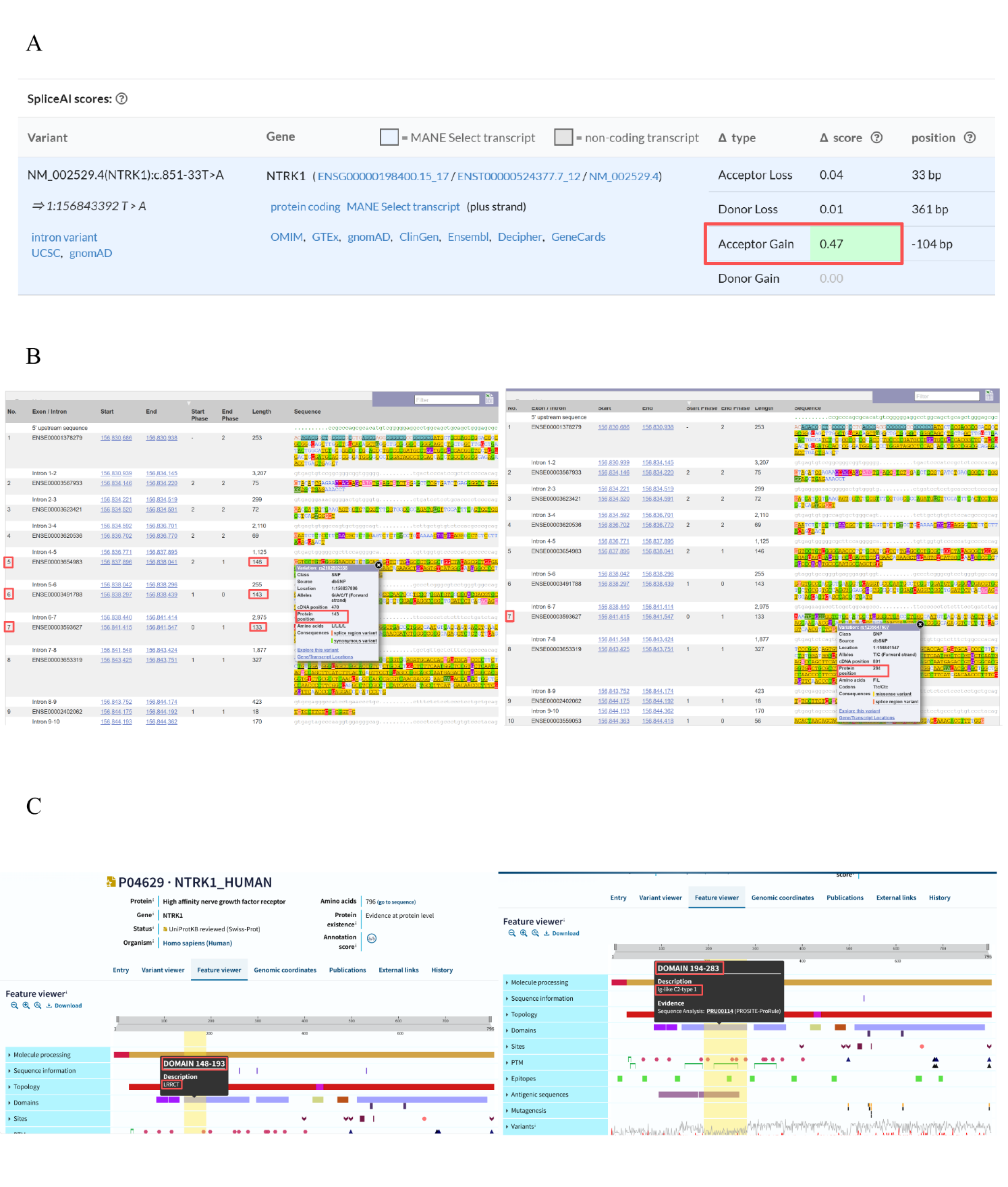


**Supplementary Figure 1.** Bioinformatic evaluation of the compound heterozygous mutations in the *NTRK1* gene (NM_002529.4). (A) Splicing prediction analysis using SpliceAI for the maternal intronic variant (c.851-33T>A), yielding a Delta Score for Acceptor Gain (DS_AG) of 0.47. (B) Genomic and transcript mapping from the Ensembl database. Red boxes highlight exons 5, 6, and 7 (encoding amino acid residues 143–284), which correspond to the paternal deletion region. (C) Functional domain architecture of the TrkA protein (UniProt). The red dashed box indicates the functional regions affected by the mutations, specifically encompassing the leucine-rich repeat C-terminal (LRRCT) domain and the immunoglobulin-like (Ig-like C2-type 1) domain.
